# Improving maternal mental health through postnatal services use for south sudanese mothers and their babies living in nguynyel refugee camp in gambella, Ethiopia

**DOI:** 10.1192/j.eurpsy.2021.356

**Published:** 2021-08-13

**Authors:** K. Le Roch, M. Lasater, G. Woldeyes, A. Solomon-Osborne, X. Phan, C. Bizouerne, S. Murray

**Affiliations:** 1 Mental Health And Care Practices, Gender And Protection, Action contre la Faim, Paris, France; 2 Mental Health And Care Practices, Action contre la Faim, Paris, France; 3 Department Of Mental Health, Johns Hopkins Bloomberg School of Public Health, Baltimore, United States of America; 4 Mental Health And Care Practices, Gender And Protection, Action Against Hunger, Addis-Ababa, Ethiopia

**Keywords:** Refugees, Process evaluation, Ethiopia, Maternal mental health

## Abstract

**Introduction:**

Poor maternal mental health during the perinatal period leads to serious complications, especially in humanitarian settings where both mothers and children have often been exposed to multiple stressful events. In those contexts, culturally relevant mental health and psychosocial interventions are required to support mother-infant dyads and ultimately to alleviate potential negative outcomes on child’s health and development.

**Objectives:**

This study aims at assessing the use of postnatal services by mothers and infants under 2 and its impact on maternal mental health.

**Methods:**

A process evaluation of Baby Friendly Spaces (BFS) program was conducted in Nguynyel refugee camp (Ethiopia) and a prospective quantitative assessment was administered to lactating women at baseline and endline (2 months later) to measure maternal functional impairment (WHODAS 2.0), general psychological distress (Kessler scale-K6); depression symptoms (Patient Health Questionnaire-PHQ9) and post-traumatic stress symptoms (PTSD Checklist-PCL-6).

**Results:**

201 lactating women and their babies were enrolled between October 2018 and March 2019. Statistically significant reductions were observed in all mental health outcomes at follow-up. Total mean scores decrease by 19% (p<0.001) for general psychological distress and posttraumatic stress, by 23% (p<0.001) for the depression and by 15% (p<0.001) for the functional impairment. Examination of the compliance to the services revealed that mothers who dropped out early had statistically significantly lower depression scores (p=0.01), and functional impairment scores (p<0.001) than mothers who stayed in the program.

**Conclusions:**

The integration of maternal mental health interventions within perinatal services is challenging but essential for identifying and treating maternal common mental disorders.

**Disclosure:**

No significant relationships.

